# Identifiability of a Binomial Synapse

**DOI:** 10.3389/fncom.2020.558477

**Published:** 2020-09-30

**Authors:** Camille Gontier, Jean-Pascal Pfister

**Affiliations:** ^1^Department of Physiology, University of Bern, Bern, Switzerland; ^2^Institute of Neuroinformatics and Neuroscience Center Zurich, University of Zurich/ETH Zurich, Zurich, Switzerland

**Keywords:** model selection, practical identifiability, structural identifiability, binomial, synapse

## Abstract

Synapses are highly stochastic transmission units. A classical model describing this stochastic transmission is called the binomial model, and its underlying parameters can be estimated from postsynaptic responses to evoked stimuli. The accuracy of parameter estimates obtained via such a model-based approach depends on the identifiability of the model. A model is said to be structurally identifiable if its parameters can be uniquely inferred from the distribution of its outputs. However, this theoretical property does not necessarily imply practical identifiability. For instance, if the number of observations is low or if the recording noise is high, the model's parameters can only be loosely estimated. Structural identifiability, which is an intrinsic property of a model, has been widely characterized; but practical identifiability, which is a property of both the model and the experimental protocol, is usually only qualitatively assessed. Here, we propose a formal definition for the practical identifiability domain of a statistical model. For a given experimental protocol, this domain corresponds to the set of parameters for which the model is correctly identified as the ground truth compared to a simpler alternative model. Considering a model selection problem instead of a parameter inference problem allows to derive a non-arbitrary criterion for practical identifiability. We apply our definition to the study of neurotransmitter release at a chemical synapse. Our contribution to the analysis of synaptic stochasticity is three-fold: firstly, we propose a quantitative criterion for the practical identifiability of a statistical model, and compute the identifiability domains of different variants of the binomial release model (uni or multi-quantal, with or without short-term plasticity); secondly, we extend the Bayesian Information Criterion (BIC), a classically used tool for model selection, to models with correlated data (which is the case for most models of chemical synapses); finally, we show that our approach allows to perform data free model selection, i.e., to verify if a model used to fit data was indeed identifiable even without access to the data, but having only access to the fitted parameters.

## 1. Introduction

Model selection is highly relevant to neuroscience, as neurons, dendrites, and synapses can be represented by models with different levels of complexity and abstraction. When it comes to fitting recorded data, predicting the output of a system to a given stimulus, or making sense of an observed phenomenon, several possible models can be used: this raises the question of what makes a good model. Finding the correct model is a crucial issue in studying the brain.

Firstly, a good model needs to be sufficiently complex to account for observed data, while being simple enough to generalize to future observations. Competing models are typically compared based on their ability to fit an observed data set, while being penalized for their complexity (or number of free parameters) to avoid overfitting. Different model selection tools (Bayesian Information Criterion, Akaike Information Criterion,.) are classically used to determine which model is the best one to fit a data set (Daw et al., 2011).

Secondly, models also differ in their nature, and can be classified as phenomenological, normative, or biophysical. On the one hand, purely phenomenological models are useful for relating the output of a system to its input, and can provide a computationally efficient way to make prediction. However, as they are solely based on the empirical relation between the input and the output of the system, and not on its inner biological principles, they lack interpretability. On the other hand, normative and biophysical models can be computationally challenging to fit on data, but are more realistic. In a normative approach, the output of a system is computed from an objective function which models its high-level functions and principles. As opposed to this top-down approach, biophysical models aim at precisely describing the low-level biological components of the system. An interesting property of these biophysical models is that their parameters correspond to real physical quantities: when the parameters of a system cannot be measured directly, they can be estimated by fitting a corresponding biophysical model on recorded output data of the system, a procedure known as model-based inference. By computing the likelihood of the data as a function of the parameters, it is possible to follow a maximum-likelihood approach to obtain a point estimate of the parameters (Barri et al., 2016), or to compute the full posterior distribution over them (Bird et al., 2016).

Such a parameter inference requires that the model used is identifiable. Structural (i.e., model-based) identifiability is a property of the model, regardless of experimental results. In a structurally identifiable system, the dimension of the output is sufficiently high with respect to the dimension of the parameters vector to uniquely define it: the parameters can be non-ambiguously inferred if the complete distribution of the output is known. Structural identifiability has been widely studied in many fields of physics and biology (Raue et al., 2009, 2011; Komorowski et al., 2011; Koyama, 2012; Hines et al., 2014), and different criteria exist to assess the structural identifiability of a model (Massonis and Villaverde, 2020).

This theoretical property is not equivalent to practical (i.e., experiment-based) identifiability, which is a property of both the model and the experimental protocol: a model which is structurally identifiable might lead to a poor practical identifiability of parameters if data points are noisy or scarce. The accuracy of model-based methods for inferring the values of parameters depends on the experimental protocol used to record the data, as observations need to be sufficiently informative to allow a correct estimation of the parameters. Contrary to structural identifiability, a quantitative criterion is lacking for practical identifiability, which is usually only qualitatively assessed. Non-practical identifiability refers to regimes in which parameters can only be loosely estimated; but one would need to define what does “loose” mean. Such a definition could be intrinsic to the model: a model could be considered as practically identifiable given a certain experimental protocol if the expected variance of its parameters' estimate is below a threshold. But this threshold would need to be arbitrarily defined. Here, we propose an extrinsic yet non-arbitrary definition of practical identifiability, by transforming a model identifiability problem into a model selection problem.

A model is said to be practically identifiable when its parameters can be correctly inferred given a certain experimental protocol. But, as explained previously, different possible models can be fitted on a data set. Recorded data need to be sufficiently informative not only to give a correct estimate of the parameters of a model, but also to select the correct model (i.e., the model from which they have been generated). We argue that a model is practically identifiable if and only if it is also correctly identified as the model providing the best fit to the data. For a given experimental protocol, we define the practical identifiability domain of a statistical model as the set of parameters for which the model is correctly identified as the ground truth compared to a simpler alternative submodel.

Our proposed definition of practical identifiability can be applied to any setting where submodels or a nested family of models can be defined. Here, we apply it to the particular problem of estimating the parameters of a chemical synapse. A classical biophysical model used to describe the stochastic release of neurotransmitter at chemical synapses is called the binomial model (Katz, 1969), for which different variants of increasing complexity (in term of the number of free parameters) can be considered.

Different model-based approaches have been proposed (Bykowska et al., 2019) for obtaining an accurate estimate of the parameters describing a synapse (namely, its number of independent release sites, their release probability upon the arrival of a presynaptic spike, the quantum of current elicited by one release event, etc.) These parameters cannot be measured directly, but can be inferred using excitatory postsynaptic currents (EPSCs^1^) recorded on the post-synaptic side and elicited by experimental stimulation of the presynaptic cell. By measuring their values before and after a stimulation protocol, it is possible to study the mechanisms and loci of synaptic plasticity (Costa et al., 2015, 2017a,b) and homeostasis (Davis and Müller, 2015; Wentzel et al., 2018). On a more theoretical level, a correct inference of synaptic parameters is necessary to study the computational role of synaptic stochasticity (Levy and Baxter, 2002; Guo and Li, 2012). Finally, an accurate inference of synaptic parameters would allow to clarify the role of synaptic transmission in different diseases (Van Spronsen and Hoogenraad, 2010), such as mental retardation (Pfeiffer and Huber, 2009), schizophrenia (Stephan et al., 2006), Parkinson's disease (Calabresi et al., 2006), autism (Südhof, 2008), Alzheimer's disease (Selkoe, 2002), compulsive behavior (Welch et al., 2007), and addiction (Kauer and Malenka, 2007).

Our contribution to the analysis of synaptic stochasticity is three-fold. Firstly, we propose a definition for the practical identifiability of a model of synaptic transmission, and compute the identifiability domains of different variants of the binomial release model. Besides, we observe that model selection criteria are classically derived by assuming that recorded data are not correlated, which does not hold for most models of chemical synapse. We extend the Bayesian Information Criterion (BIC), a classically used tool for model selection, to models with correlated data. Finally, a proper description of the model selection step is often missing in studies where a model based-approach is used to infer synaptic parameters. We show that our approach allows to perform *data free* model selection, i.e., to verify if a model used to fit data was indeed identifiable even if a proper model selection step had not been performed.

## 2. Methods

### 2.1. Binomial Models of Neurotransmitter Release

#### 2.1.1. The Classical Binomial Model

The quantal nature of synaptic transmission was first unveiled in Del Castillo and Katz (1954), in which the authors observed that the postsynaptic responses to presynaptic stimulations were all multiples of a small unit of current. They explained how the total response is built up of several of these units, or quanta, each of them arising from a single presynaptic release event. Upon the arrival of an action potential in the presynaptic terminal, vesicles are released with a given probability *p*. The binomial model (Katz, 1969) assumes that there are *N* independent release sites and that for each site the release probability *p* is identical. Therefore, the number of released vesicles *k*_*i*_ after spike *i* is distributed according to a binomial distribution. This model further assumes that each vesicle release gives rise to a quantal current *q*, such that the overall excitatory postsynaptic current is given by *e*_*i*_ = *qk*_*i*_ + ϵ, where ϵ models a measurement noise typically drawn from a normal distribution with variance σ^2^. Under the binomial model described by its parameters *N*, *p*, *q*, and σ, the distribution of EPSCs is given by

$$p\left(e_{i}\right)=\overset{N}{\underset{k_{i}=0}{\sum }}p\left(e_{i}|k_{i}\right)p\left(k_{i}\right)$$

where *k*_*i*_ follows a binomial distribution with parameters *N* and *p*, and *e*_*i*_ conditioned on *k*_*i*_ follows a normal distribution with mean *qk*_*i*_ and variance σ^2^. Postsynaptic responses are characterized by their mean *Npq* and their variance *q*^2^*Np*(1 − *p*) + σ^2^. A first feature of synaptic transmission is thus its stochasticity. Due to different sources of noise, such as probabilistic vesicles release or recording noise, postsynaptic recordings exhibit trial-to-trial variability.

#### 2.1.2. Full Model of Synaptic Transmission

Although this simple binomial model accounts for synaptic stochasticity, it does not allow to model its dynamics: postsynaptic responses do not only depend on the parameters of the synapse, but also on its previous activity. On the one hand, successive presynaptic stimulations within a short time interval will lead to a depletion of the readily-releasable vesicle pool, and hence to reduced successive postsynaptic responses, a phenomenon known as short-term depression. This can be modeled by assuming that the number of available vesicles at time *i* is *n*_*i*_ ≤ *N* (while the simplified binomial model described above assumes that all vesicles are readily releasable, and hence *n*_*i*_ = *N*). On the other hand, successive stimulations will gradually increase the presynaptic calcium concentration, and hence the release probability, which is called short-term facilitation.

Short-term depression and facilitation can be modeled using the Tsodyks-Markram model (Tsodyks et al., 1998; Costa et al., 2015). It consists in two ordinary differential equations, which model the proportion of available vesicles *r*_*i*_ and the release probability *u*_*i*_ at time *i*. *r*_*i*_ is reduced by an amount *u*_*i*_*r*_*i*_ after each presynaptic spike, and recovers back to 1 with a depression time constant τ_*D*_ between each spike. Similarly, *u*_*i*_ is increased by an amount *p*(1 − *u*_*i*_), and decays back to *p* (its baseline value) with a facilitation time constant τ_*F*_. Different values of the parameters *p*, τ_*D*_, and τ_*F*_ allow to represent different synaptic dynamics (either depression, facilitation, or no plasticity at all).

However, such a deterministic approach to short-term plasticity only allows to model averages, and neglects correlations between successive postsynaptic responses. In recent studies (Barri et al., 2016; Bird et al., 2016), models of synapses incorporating both short-term plasticity and binomial models of vesicles release and refill have been proposed. In these models, the release probability *u*_*i*_ evolves according to the equation of the Tsodyks-Markram model, while each vesicle refills with a probability 1 − exp( − Δ_*t*_*i*__/τ_*D*_), where Δ_*t*_*i*__ is the time interval between two successive presynaptic stimulations. This approach allows to represent both the stochasticity and the dynamics of neurotransmitter release, and to compute the likelihood of a set of recorded data D given the parameters θ and the presynaptic stimulation protocol Ψ.

**Figure 1:**
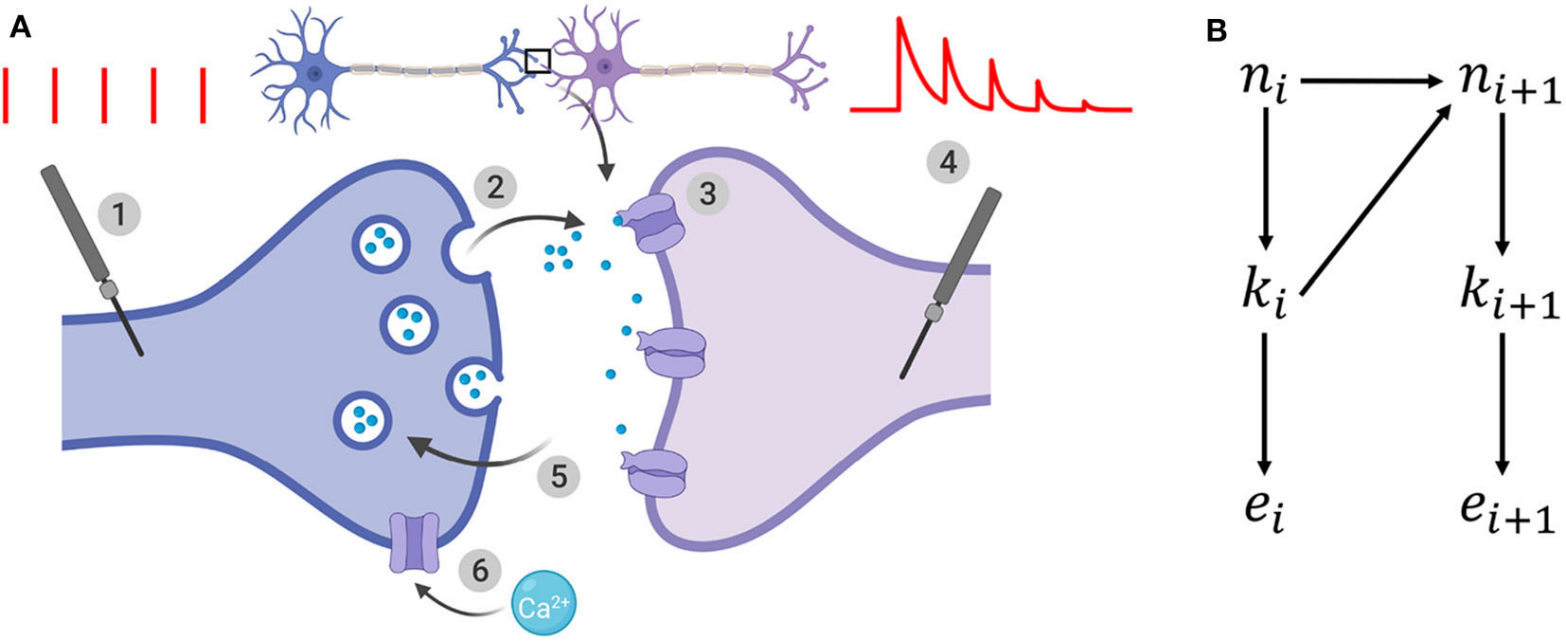
**(A)** Illustration of the binomial model. (1): the presynaptic button is artificially stimulated. Red vertical bars show 5 presynaptic spikes with a constant interspike interval. (2): these evoked stimuli lead to neurotransmitter release. After spike *i*, *k*_*i*_ vesicles (out of the *n*_*i*_ vesicles from the readily-releasable vesicle pool) release their neurotransmitter with a probability *u*_*i*_. (3): neurotransmitter bind to receptors and elicit a postsynaptic current. A single release event triggers a quantal response of amplitude *q*. (4): the recorded postsynaptic response after spike *i* is the sum of the effects of the *k*_*i*_ release events. EPSCs correspond to the amplitude of each peak of the postsynaptic response to a presynaptic spike. (5): out of the *N* release sites, only *n*_*i*_ are in the readily-releasable vesicle pool at the moment of spike *i*. After releasing, vesicles recover with a time constant τ_*D*_ which determines short-term depression. (6): in the same time, each spike increases the calcium concentration in the presynaptic button, and hence increases the release probability *u*_*i*_. This short-term facilitation is characterized by a time constant τ_*F*_. **(B)** Generative model for the dynamical binomial model where *n*_*i*_ is the number of ready releasable vesicles and *k*_*i*_ is the number of released vesicles at time *i*. *e*_*i*_ is the EPSC amplitude at time *t*_*i*_.

We consider a model of chemical synapse which encompasses both short-term depression (STD) and facilitation (STF) (Barri et al., 2016; Bird et al., 2016). Its parameters are (Figure 1A):

- *N*: the number of independent release sites [-]
- *p*: their initial release probability [-]
- σ: the recording noise. It encompasses both the noise coming from the experimental apparatus (thermal noise of the amplifier, electric line noise, etc.) and from the recordings per se (such as fluctuations in the membrane potential of the cell) [A]
- *q*: the quantum of current elicited by one release event [A]^2^
- τ_*D*_: the time constant of vesicles refilling, and hence of short-term depression [s]
- τ_*F*_: the time constant of *Ca*^2+^ dynamics, and hence of short-term facilitation [s]

which defines a vector θ = (*N, p, q*, σ, τ_*D*_, τ_*F*_). A probability conditioned on a parametrization θ is written *p*_θ_.

**n** = {*n*_*i*_}_1 ≤ *i* ≤ *T*_, **k** = {*k*_*i*_}_1 ≤ *i* ≤ *T*_, and $D={\left\{e_{i}\right\}}_{1\leq i\leq T}$ represent respectively the number of available vesicles at the moment of spike *i*, the number of vesicles released after spike *i*, and the i-th recorded EPSC (Figure 1B). The experimental protocol Ψ = {*t*_1_, *t*_2_, …, *t*_*T*_} encompasses the times of presynaptic stimulation: the time of the i-th spike is written *t*_*i*_ and Δ*t*_*i*_ = *t*_*i*_ − *t*_*i*−1_. For simplicity, we will drop the dependency on Ψ from the notations of probabilities.

The probability of recording D is computed as the marginal of the joint distribution of the observations D and the hidden variables **n** and **k**:

(1) $$\begin{array}{l} p_{\theta }\left(D,\text{n},\text{k}\right)=p_{\theta }\left(e_{1}|k_{1}\right)p_{\theta }\left(k_{1}|n_{1}\right)p_{\theta }\left(n_{1}\right)\\ \overset{T}{\underset{i=2}{\prod }}p_{\theta }\left(e_{i}|k_{i}\right)p_{\theta }\left(k_{i}|n_{i}\right)p_{\theta }\left(n_{i}|n_{i-1},k_{i-1}\right) \end{array}$$

where *p*_θ_(*e*_*i*_ | *k*_*i*_) is the emission probability, i.e., the probability to record *e*_*i*_ knowing that *k*_*i*_ vesicles released neurotransmitter and assuming a normally distributed recording noise^3^:

(2) $$\begin{array}{l} p_{\theta }\left(e_{i}|k_{i}\right)=\frac{1}{\sigma \sqrt{2\pi }}\exp\left(-\frac{{\left(e_{i}-qk_{i}\right)}^{2}}{2\sigma^{2}}\right) \end{array}$$

*p*_θ_(*k*_*i*_ | *n*_*i*_) is the binomial distribution and represents the probability that, given *n*_*i*_ available vesicles, *k*_*i*_ of them will indeed release neurotransmitter:

(3) $$\begin{array}{l} p_{\theta }\left(k_{i}|n_{i}\right)=\left(\begin{array}{c} n_{i}\\ k_{i} \end{array}\right)u_{i}^{k_{i}}{\left(1-u_{i}\right)}^{n_{i}-k_{i}} \end{array}$$

where the release probability *u*_*i*_ evolves as

(4) $$\begin{array}{l} u_{i}=p+u_{i-1}\left(1-p\right)\exp\left(-\frac{\Delta t_{i}}{\tau_{F}}\right) \end{array}$$

with *u*_1_ = *p*. *p*_θ_(*n*_*i*_ | *n*_*i*−1_, *k*_*i*−1_) represents the process of vesicles refilling. During the time interval Δ*t*_*i*_, each empty vesicle can refill with a probability *I*_*i*_:

(5) $$\begin{array}{l} p_{\theta }\left(n_{i}|n_{i-1},k_{i-1}\right)=\left(\begin{array}{c} N-n_{i-1}+k_{i-1}\\ n_{i}-n_{i-1}+k_{i-1} \end{array}\right){\;I}_{i}^{n_{i}-n_{i-1}+k_{i-1}}{\left(1-I_{i}\right)}^{N-n_{i}} \end{array}$$

with

(6) $$\begin{array}{l} I_{i}=1-\exp\left(-\frac{\Delta t_{i}}{\tau_{D}}\right) \end{array}$$

It is usually assumed that, at the beginning of the experiment, all release sites are filled, and hence that *n*_1_ = *N* (Barri et al., 2016; Bird et al., 2016).

### 2.2. Models, Submodels, and Nested Families

**Definition 2.1. Model**. For a given data set D and experimental protocol Ψ, a model M is defined as a triplet M={Θ,π,L} where Θ is the space of parameters θ ∈ Θ, π is the prior for the parameters π(θ)=p(θ|M), and L is the likelihood of the parameters L(θ|D)=p(D|θ,M,Ψ).

**Examples:** Different models can be derived from Equations (1) to (6). We consider four models of decreasing complexity:

Model $M_{3}$ is the full model with both STD and STF. Its six parameters are *N, p, q*, σ, τ_*D*_, and τ_*F*_, and hence $\Theta_{3}={\mathbb{N}}^{*}\;\times \;\left[0,1\right]\;\times {\left({\mathbb{R}}_{+}\right)}^{4}$. Its likelihood function $L_{3}$ is obtained by marginalizing out the hidden variables **n** and **k**:

(7) $$\begin{array}{l} L_{3}\left(\theta |D\right)=\underset{\text{n},\text{k}}{\sum }p_{\theta }\left(D,\text{n},\text{k}\right) \end{array}$$

where $p_{\theta }\left(D,\text{n},\text{k}\right)$ is given by Equation (1).

Model $M_{2}$ has only short-term depression (and no short-term facilitation). Its five parameters are *N, p, q*, σ, and τ_*D*_, and hence $\Theta_{2}={\mathbb{N}}^{*}\;\times \;\left[0,1\right]\;\times {\left({\mathbb{R}}_{+}\right)}^{3}$. Its likelihood $L_{2}$ can be derived from (7) by further assuming that *u*_*i*_ is a constant equal to *p*.

Model $M_{1}$ shows no short-term plasticity at all, and can be derived from model $M_{2}$ by further assuming that *I*_*i*_ (defined in 6) is a constant equal to 1 (and hence *n*_*i*_ = *N*). Its four parameters are *N, p, q*, and σ, and hence $\Theta_{1}={\mathbb{N}}^{*}\;\times \;\left[0,1\right]\;\times {\left({\mathbb{R}}_{+}\right)}^{2}$. In this setting, data points are independent and (7) becomes

(8) $$\begin{array}{l} L_{1}\left(\theta |D\right)=\overset{T}{\underset{i=1}{\prod }}\left(\overset{N}{\underset{k_{i}=0}{\sum }}p_{\theta }\left(e_{i}|k_{i}\right)p_{\theta }\left(k_{i}\right)\right) \end{array}$$

with $p_{\theta }\left(k_{i}\right)=\left(\begin{array}{c} N\\ k_{i} \end{array}\right)p^{k_{i}}{\left(1-p\right)}^{N-k_{i}}$ being the binomial distribution;

Model $M_{0}$ is a Gaussian model, in which EPSCs are simply generated from a normal distribution parameterized by its mean and variance. Its two parameters are μ and σ^2^, and hence Θ_0_ = ℝ × ℝ_+_. Its likelihood $L_{0}$ is simply

(9) $$\begin{array}{l} L_{0}\left(\theta |D\right)=\overset{T}{\underset{i=1}{\prod }}p_{\theta }\left(e_{i}\right) \end{array}$$

with $p_{\theta }\left(e_{i}\right)=\frac{1}{\sigma \sqrt{2\pi }}\exp\left(-\frac{{\left(e_{i}-\mu \right)}^{2}}{2\sigma^{2}}\right)$.

To ensure the completeness of the definition of the models, we will assume for each parameter θ a uniform prior between two values θ_min_ and θ_max_ (Bird et al., 2016). Note however that the approximate identifiability domain defined in (17) does not depend on the prior.

**Definition 2.2. Submodels**. Although ubiquitous in statistics (as in the likelihood-ratio test or Pearson's chi-squared test), the notion of submodels (or nested models) is rarely formally defined in the literature (Edwards and MacCallum, 2012). It is usually said that $M_{0}$ is a submodel of $M_{1}$ (or is nested within $M_{1}$) if $M_{0}$ can be obtained by constraining the parameters of $M_{1}$ (Gottman, 1995). We propose the following formal definition, that encompasses the space of parameters, their priors, and their likelihood.

$M_{0}=\left\{\Theta_{0},\pi_{0},L_{0}\right\}$ is said to be a submodel of $M_{1}=\left\{\Theta_{1},\pi_{1},L_{1}\right\}$ if

1. Θ_0_ ⊂ Θ_1_ (i.e. the parameters of $M_{0}$ also appear in $M_{1}$)
2. $\pi_{0}\left(\theta_{0}\right)=\int_{\Theta_{1}\backslash \Theta_{0}}\pi_{1}\left(\theta_{0},\tilde{\theta }\right)d\tilde{\theta },\;\forall \theta_{0}\in \Theta_{0}$ (i.e. $M_{0}$ and $M_{1}$ share the same priors for the parameters they have in common)
3. $\forall \theta_{0}\in \Theta_{0},\exists \tilde{\theta }$ s.t. $p\left(D|\theta_{0},M_{0}\right)=p\left(D|\left(\theta_{0},\tilde{\theta }\right),M_{1}\right)$ with $\left(\theta_{0},\tilde{\theta }\right)\in \Theta_{1}$ (i.e., $M_{0}$ can be retrieved from $M_{1}$ by constraining its parameters).

We use the notation $M_{0}≼M_{1}$.

**Examples:** The model $M_{2}$ with only short-term depression is a submodel of $M_{3}$ (which accounts for both depression and facilitation). Indeed, they have the parameters *N*, *p*, *q*, σ, and τ_*D*_ in common, and $M_{2}$ can be retrieved from $M_{3}$ by constraining $\tau_{F}\overset{}{\underset{}{\to }}0$. Similarly, the model without STP $M_{1}$ is a submodel of $M_{2}$ where $\tau_{D}\overset{}{\underset{}{\to }}0$; and the uni-quantal model $M_{0}$ is a submodel of the multi-quantal model $M_{1}$ where *p* = 1 and μ = *Nq*.

We propose the following definitions to characterize the nestedness of a family of models:

** Definition 2.3. Nested family**. $F=\left\{M_{0},M_{1},\ldots ,M_{n}\right\}$ is said to be a nested family if

$$M_{i}≼M_{j},\;\forall 0\leq i\leq j\leq n$$

### 2.3. Structural Identifiability

** Definition 2.4. Structural identifiability domain**. Consistently with Raue et al. (2009) and Massonis and Villaverde (2020), let the structural identifiability domain Θ_S_ of a model M={Θ,π,L} be defined as:

(10) $$\begin{array}{l} \Theta_{\text{S}}=\left\{\theta \in \Theta \;|\;\forall \theta^{\prime }\in \Theta ,\;\theta \neq \theta^{\prime }\Leftrightarrow p\left(D|\theta ,M\right)\neq p\left(D|\theta^{\prime },M\right)\right\} \end{array}$$

Similarly, M is said to be structurally identifiable if Θ = Θ_S_.

**Figure 2:**
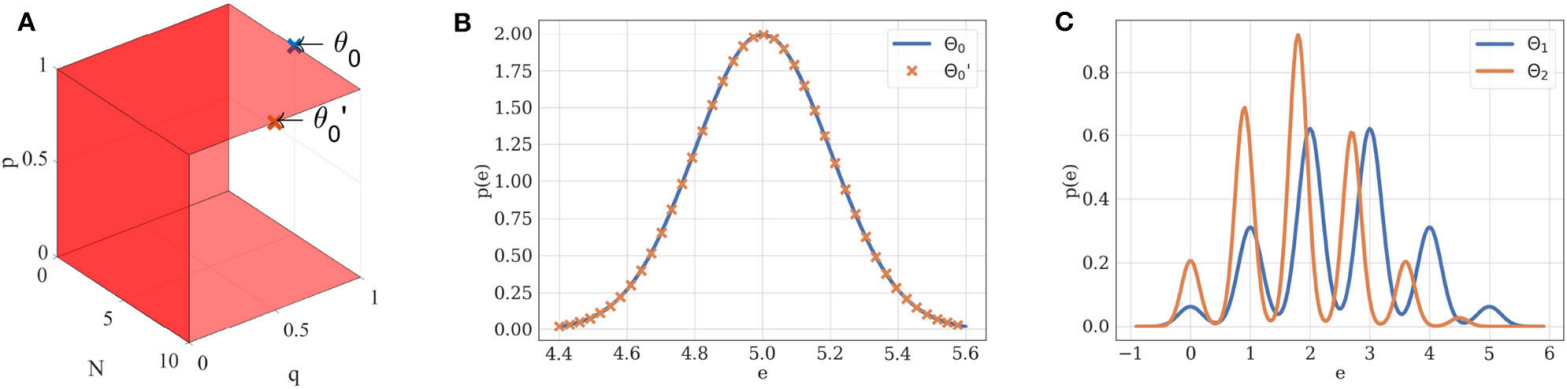
**(A)** Structural identifiability domain of the binomial model $M_{1}$. This model, parameterized by θ = (*N, p, q*, σ), is structurally identifiable if and only if *N* ≠ 0, *p* ≠ 0, *p* ≠ 1, and *q* ≠ 0. These conditions are represented by the red hyperplanes in the *N* × *p* × *q* domain. **(B)** Two different sets of parameters θ_0_ and $\theta_{0}^{\prime }$ may lead to the same distribution of observations if taken out of the structural identifiability domain. If *p* = 1, the distribution of EPSCs under model $M_{1}$ follows a Gaussian law of variance σ^2^ and mean *Nq*: different combinations of *N* and *q* can thus ambiguously describe it. Blue distribution: *N* = 5, *p* = 1, *q* = 1, σ = 0.2. Orange distribution: *N* = 10, *p* = 1, *q* = 0.5, σ = 0.2. **(C)** Two different sets of parameters θ_1_ and θ_2_ will lead to different distributions when taken within the structural identifiability domain of $M_{1}$: its distribution is uniquely defined by its parameters. Blue distribution: *N* = 5, *p* = 0.5, *q* = 1, σ = 0.2. Orange distribution: *N* = 5, *p* = 0.4, *q* = 0.9, σ = 0.15.

If θ is in the structural identifiability domain of M, it can be uniquely identified from p(D|θ,M). For instance, a Gaussian distribution of mean μ and variance σ^2^ is uniquely defined by its parameters θ = (μ, σ^2^). Its structural identifiability domain is thus $\Theta_{\text{S}}=\mathbb{R}\times {\mathbb{R}}^{+}$. Similarly, if *N* ≠ 0, *p* ≠ 0, *p* ≠ 1, and *q* ≠ 0, the probability density of EPSCs under the binomial model without short-term plasticity $M_{1}$ is structurally identifiable if we restrict Θ_1_ to ${\mathbb{N}}^{*}\;\times \;]0,1[\;\times {\left({\mathbb{R}}_{+}^{*}\right)}^{2}$ (Figure 2).

### 2.4. Informative Domain

In some regimes, parameters may not be precisely inferred from observations, even though the model is otherwise structurally identifiable. Indeed, in practice we usually only have access to a finite number of (possibly noisy) observations. *Practical* identifiability thus differs from the *structural* identifiability defined in section 2.3.

A definition for the practical identifiability of a parameter has previously been proposed in Raue et al. (2009), along with an approach for detecting practical non-identifiabilities based on the profile likelihood (Venzon and Moolgavkar, 1988; Murphy and Van der Vaart, 2000). The authors first define the likelihood-based confidence intervals for the estimator $\hat{\theta_{i}}$ of the i-th parameter of a model M:

$$C_{i,\Delta }=\left\{\theta_{i}\;|\;L\left(\hat{\theta_{i}}|D\right)-L\left(\theta_{i}|D\right)<\Delta \right\}$$

where

$$L\left(\theta_{i}|D\right)=\underset{\theta_{j\neq i}}{\max}L\left(\theta |D\right)$$

for a given threshold Δ. Then, they propose the following definition: *A parameter estimate* $\hat{\theta_{i}}$ *is practically non-identifiable, if the likelihood-based confidence region is infinitely extended in increasing and/or decreasing direction of* θ_*i*_*, although the likelihood has a unique minimum for this parameter*, meaning that the decrease in likelihood compared to the optimal parameters estimate stays below the threshold Δ in direction of θ_*i*_. When plotting the likelihood as a function of the parameters, practical non-identifiability can be seen as an infinitely extended flat valley, in which the decrease in likelihood stays below Δ. The authors also describe an algorithm for computing the profile likelihood and hence detecting such practical non-identifiabilities: *Structural non-identifiable parameters are characterized by a flat profile likelihood. The profile likelihood of a practically non-identifiable parameter has a minimum, but is not excessing a threshold* Δ *for increasing and/or decreasing values of* θ_*i*_ (see Figure 3 in Raue et al., 2009).

**Figure 3:**
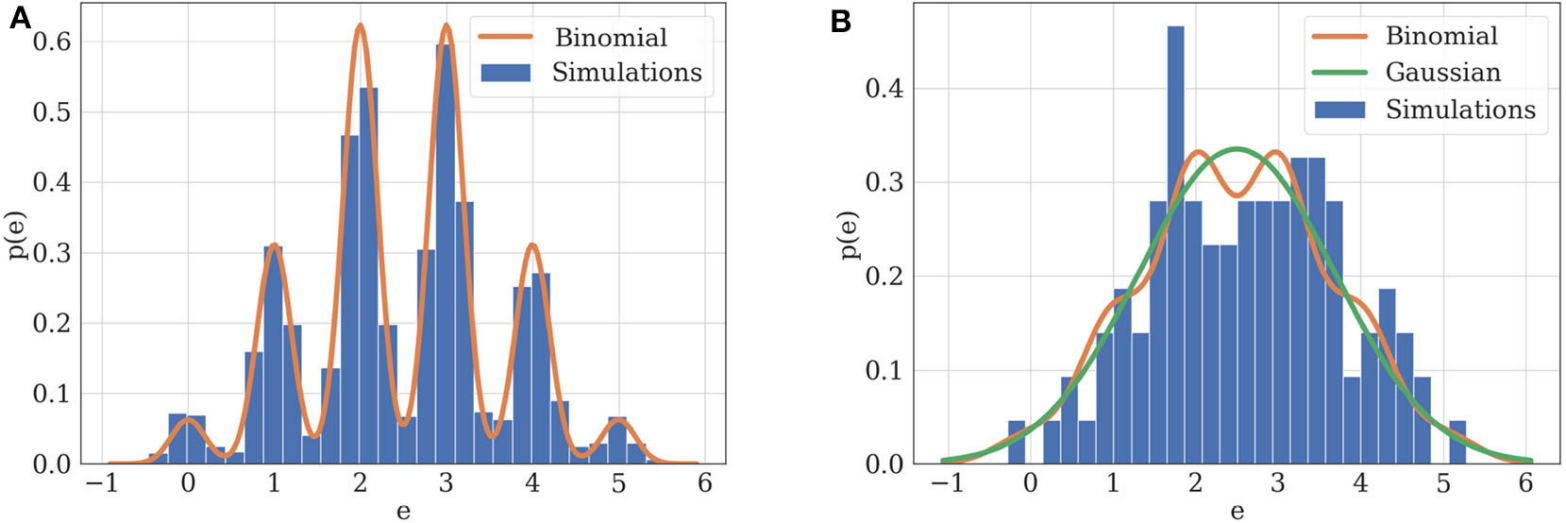
**(A)** Illustration of practical identifiability. Orange: theoretical distribution for model $M_{1}$ parameterized with *N* = 5, *p* = 0.5, *q* = 1, σ = 0.2. Blue: histogram of 2,000 simulated EPSCs generated using the same parameters. Parameters can be precisely inferred from the observations, which fit their theoretical distribution. **(B)** Illustration of practical non-identifiability. Blue: histogram of 100 simulated EPSCs with *N* = 5, *p* = 0.5, *q* = 1, σ = 0.4. Due to the small number of data points and high recording noise σ, the binomial parameters can only be loosely estimated, which is characterized by the fact that a Gaussian distribution (green) will provide a better fit to the data than a binomial distribution (orange).

An important limitation of this definition is to be data-dependent: it only holds for a specific set of recorded data D. Indeed, likelihood-based confidence intervals, and hence practical identifiability, are defined with respect to a certain data set D, and may thus vary for different realizations of the experiment. However, an identifiability criterion can be made data-independent by averaging it over all possible realizations of D, i.e., by computing its expectation with respect to the distribution $p\left(D|\theta^{*},M,\text{Ψ}\right)$. Such an averaged criterion would correspond to the *a priori* expected identifiability before a specific D is recorded.

Practical information about θ is a function of the experimental protocol Ψ: for a given Ψ, the informative domain Θ_I_(Ψ) of a model M could be defined based on the variance of the estimator. For instance, in a Bayesian setting, the domain Θ_I_ (Ψ) could be the set of parameters for which the expected informativeness of the posterior distribution of the parameters (measured as the Kullback-Leibler divergence between the posterior and the prior) is above a threshold Δ:

(11) $$\begin{array}{l} \Theta_{\text{I}}\left(\text{Ψ}\right)=\left\{\theta^{*}\in \Theta \;|\;{\langle D_{KL}\left(p\left(\theta |D,M,\text{Ψ}\right)\;||\;p\left(\theta |M\right)\right)\rangle }_{p\left(D|\theta^{*},M,\text{Ψ}\right)}\geq \Delta \right\} \end{array}$$

Although data-independent, this definition suffers from the same limitation as the one proposed in Raue et al. (2009): it requires to set a specific threshold Δ. Instead of defining an arbitrary criterion Δ on the possible precision of parameters estimate, we will derive our definition from a model selection argument.

### 2.5. Model Selection

In model selection, the plausibility of two competing models M={Θ,π,L} and $M^{\prime }=\left\{\Theta^{\prime },\pi^{\prime },L^{\prime }\right\}$ based on observations D can be assessed using the Bayes Factor (Kass and Raftery, 1995):

(12) $$\begin{array}{l} B_{M,M^{\prime }}\left(D\right)=\frac{p\left(D|M\right)}{p\left(D|M^{\prime }\right)}=\frac{\int_{\Theta }L\left(\theta |D\right)\pi \left(\theta \right)d\theta }{\int_{\Theta^{\prime }}L^{\prime }\left(\theta |D\right)\pi^{\prime }\left(\theta \right)d\theta } \end{array}$$

If the Bayes Factor is superior to 1, then the evidence for M is higher than the evidence for $M^{\prime }$. It is worth pointing out that the Bayes Factor will not only favor models which provide a good fit to the data, but also includes a tendency to favor simpler models, a natural form of Occam's Razor (Jefferys and Berger, 1991; MacKay and MacKay, 2003). Indeed, a complex model (i.e., a model with many independent parameters or with a broader prior for its parameters) will be able to explain a larger set of possible observed data than a simple model; but this comes at the price of spreading its likelihood over a larger set of possible outcomes. Hence, if two models fit the observed data equally well, the simpler one will be favored.

### 2.6. Proposed Definition of Practical Model Identifiability

To compute the identifiability domain of any model M compared to another model $M^{\prime }$, we introduce the Average Log Bayes Factor:

(13) $$\begin{array}{l} B_{M,M^{\prime }}\left(\theta^{*},\text{Ψ}\right)={\langle \log B_{M,M^{\prime }}\left(D\right)\rangle }_{p\left(D|\theta^{*},M,\text{Ψ}\right)} \end{array}$$

For a given parameter θ^*^ and protocol Ψ, model M is said to be practically identifiable compared to $M^{\prime }$ if $B_{M,M^{\prime }}\left(\theta^{*},\text{Ψ}\right)\geq 0$. Intuitively, the identifiability domain of M compared to $M^{\prime }$ corresponds to all the settings (parameters and protocols) for which, on average, data generated from the ground truth M will be better explained by M than by $M^{\prime }$.

In contrast to the definition in Raue et al. (2009), our proposed definition does not require to set a (possibly arbitrary) threshold Δ. Instead, it is derived from a model selection criterion. We argue that the parameters of a model M are practically identifiable if M is itself practically identifiable. In some settings (as for the nested models of chemical synapse described in section 2.1), a family of submodels might naturally arise, while the choice of a threshold Δ would be arbitrary.

Another interest of our approach is to be data-independent, while the definition proposed in Raue et al. (2009) only holds for a specific set of recorded data D. Indeed, we define practical identifiability as a data-independent and intrinsic property of the model M and experimental protocol Ψ. As the log-Bayes Factor in (13) is averaged over all possible realizations of D, it corresponds to the *a priori* expected identifiability before D is recorded. Our approach thus allows to define practical identifiability *domains*:

** Definition 2.5. Practical identifiability domain**. Consider a model M={Θ,π,L} and a submodel $M^{\prime }=\left\{\Theta^{\prime },\pi^{\prime },L^{\prime }\right\}$ of M. For a given experimental protocol Ψ, the practical identifiability domain Θ_P_ (Ψ) of M is the set of parameters θ^*^ for which it is identifiable compared to its submodel:

(14) $$\begin{array}{l} \Theta_{\text{P}}\left(\text{Ψ}\right)=\left\{\theta^{*}\in \Theta \;|\;B_{M,M^{\prime }}\left(\theta^{*},\text{Ψ}\right)\geq 0\right\} \end{array}$$

Note that in the limit where the priors π and π′ are highly peaked (i.e., $\pi \left(\theta \right)=\delta \left(\theta -\bar{\theta }\right)$ and $\pi^{\prime }\left(\theta \right)=\delta \left(\theta -{\bar{\theta }}^{\prime }\right)$), the condition $B_{M,M^{\prime }}\left(\theta^{*},\text{Ψ}\right)\geq 0$ is always satisfied due to Gibbs' inequality. In this case we have Θ_P_ (Ψ) = Θ, ∀Ψ. However, generically the condition $B_{M,M^{\prime }}\left(\theta^{*},\text{Ψ}\right)\geq 0$ is not always satisfied since p(D|M) is not equal to $p\left(D|\theta^{*},M\right)$. The latter is the probability of observing D given M and a certain parametrization θ^*^, while the former is the marginal likelihood over all parameters (12).

Two examples can illustrate this correspondence between model selection and parameter inference. Consider first the case of data recorded from $M_{1}$. If the experimental protocol is not sufficiently informative (i.e., if data are scarce or noisy), not only will the inference of synaptic parameters be poor, but a Gaussian distribution will also provide a better fit than a binomial release model to the data. Indeed, as $\left[e_{i}|k_{i}\right]\sim N\left(qk_{i},\sigma \right)$, in the absence of recording noise (i.e., if σ = 0), the distribution of EPSCs is a series of Dirac delta functions located at each multiple of the quantal size *qk* for *k* ∈ {0, 1, …, *N*}. In this ideal case, *q* is clearly identifiable (Figure 3A). However, upon addition of a recording noise of amplitude σ, EPSCs are normally distributed around *qk* for *k* ∈ {0, 1, …, *N*}, and the peaks on the histogram corresponding to each multiple of the quantal size might overlap if σ is sufficiently high with respect to *q* (Figure 3B).

Similarly, we can consider the example of a synapse which shows short-term depression (STD) with a time constant τ_*D*_ (model $M_{2}$). If the presynaptic cell is stimulated with an inter-spike intervals longer than τ_*D*_, no depression will be visible in the recorded data, and the true model with STD will not be identifiable from a simpler binomial model without STD. In the same time, it will impossible to correctly infer the value of τ_*D*_.

Our proposed definition of practical identifiability and of the identifiability domain of a model extend the landscaping technique introduced in Navarro et al. (2004) as well as the framework for testing identifiability of Bayesian models introduced in Acerbi et al. (2014). Especially, comparing the expected supports ${\langle \log p\left(D|M\right)\rangle }_{p\left(D|\theta^{*},M,\text{Ψ}\right)}$ and ${\langle \log p\left(D|M^{\prime }\right)\rangle }_{p\left(D|\theta^{*},M,\text{Ψ}\right)}$ of M and $M^{\prime }$ (given that values are averaged over ${\langle \cdot \rangle }_{p\left(D|\theta^{*},M,\text{Ψ}\right)}$) allows us to define a quantitative criterion for identifiability.

The model evidence p(D|M) in (12) is often intractable in practice for complex models, as it requires to integrate marginals for each parameter. Different methods have been proposed to approximate it: MCMC computations (Weinberg et al., 2012), Savage-Dickey method (Wagenmakers et al., 2010), supermodels (Mootoovaloo et al., 2016). A practical and time-efficient approximation of the model evidence is given by the Bayesian Information Criterion $BIC_{M}\left(D\right)$ (Schwarz et al., 1978):

(15) $$\begin{array}{l} \text{BI}{\text{C}}_{M}\left(D\right)=-2\log p\left(D|\hat{\theta },M\right)+k_{M}\log\left(\text{T}\right)\approx -2\log p\left(D|M\right) \end{array}$$

where $\hat{\theta }={arg max}_{\theta }L\left(\theta |D\right)$ is the maximum likelihood estimator (MLE) of L(θ|D), $k_{M}=dim\left(\Theta \right)$ is the number of independent parameters of M, and *T* = |Ψ| is the number of data points in D. A detailed derivation is provided in Supplementary Material. The BIC is the sum of two terms: a likelihood term $-2\log p\left(D|\hat{\theta },M\right)$ which represents the ability of the model M to explain D, and a penalty term $k_{M}\log\left(T\right)$ which favors simpler models, as explained in section 2.5.

The BIC is commonly used as an approximation of the model evidence p(D|M) in model selection: the model with the lowest BIC is preferred over the others. The main advantage of using the BIC is to transform a complex integration problem (i.e., the computation of p(D|M)) into a simpler optimization problem (i.e. the computation of $\hat{\theta }$). Besides, it allows to perform model selection without the need to specify a prior for the parameters, and is thus a popular tool for model selection (Daw et al., 2011).

As stated in Supplementary Material, the approximation ${\text{BIC}}_{M}\left(D\right)\approx -2\log\text{p}\left(D|M\right)$ is only valid under the hypothesis that data points are independent and identically distributed (i.i.d.), which is not the case for models with short-term plasticity. If data are correlated, we are left with the following approximation, which does not simplify in the general case:

(16) $$\begin{array}{l} -2\log p\left(D|M\right)\approx -2\log p\left(D|\hat{\theta },M\right)+\log\left(\left|H\left(\hat{\theta }\right)\right|\right) \end{array}$$

where $H\left(\hat{\theta }\right)$ is the Hessian matrix of -logp(D|θ,M) in Equation (15).

We emphasize that the classical definition of the BIC (15) should not be used if observations are correlated. Here, for models in which output are not independent, we use the approximation given by Equation (16), in which the term $k_{M}\log\left(T\right)$ in the BIC is replaced by $\log\left(\left|H\left(\hat{\theta }\right)\right|\right)$. In some settings, the computation of the Hessian matrix can be challenging. However, MCMC methods can be used to approximate $H\left(\hat{\theta }\right)$, even without an explicit expression for the gradient of the function (Spall, 2005). In our case, a numerical method for computing $\left|H\left(\hat{\theta }\right)\right|$ is detailed in the Supplementary Material.

Using approximation (15) in definition (14) yields the following approximation for the practical identifiability domain in case the model evidence p(D|M) in (12) is intractable:

(17) $$\begin{array}{l} {\tilde{\Theta }}_{P}(\Psi )=\{\theta^{*}\in \Theta \;|\;{\langle {\text{BIC}}_{M}(D)\rangle }_{\text{p}(D|\theta^{*},M,\Psi )}\\ \;\leq {\langle {\text{BIC}}_{M^{\prime }}(D)\rangle }_{\text{p}(D|\theta^{*},M,\Psi )}\} \end{array}$$

## 3. Results

### 3.1. Identifiability Domain of the Binomial Model Without Short-Term Plasticity

We study here the conditions under which a binomial model without short-term plasticity $M_{1}$ can be correctly identified from a Gaussian model having the same mean and variance ($M_{0}$). In order for the binomial model to be identifiable from a Gaussian quantum-less distribution, the recording noise needs to be sufficiently low compared to *q* for the peaks on the histogram of recorded EPSC to be identified. We will thus plot the identifiability domain as a function of the recording noise of amplitude σ for a fixed *q*. The identifiability domain corresponds to the points θ in the parameters space Θ_1_ for which the average BIC of $M_{1}$ over all possible outputs of $M_{1}$ parameterized with θ is lower than the average BIC of $M_{0}$.

**Figure 4:**
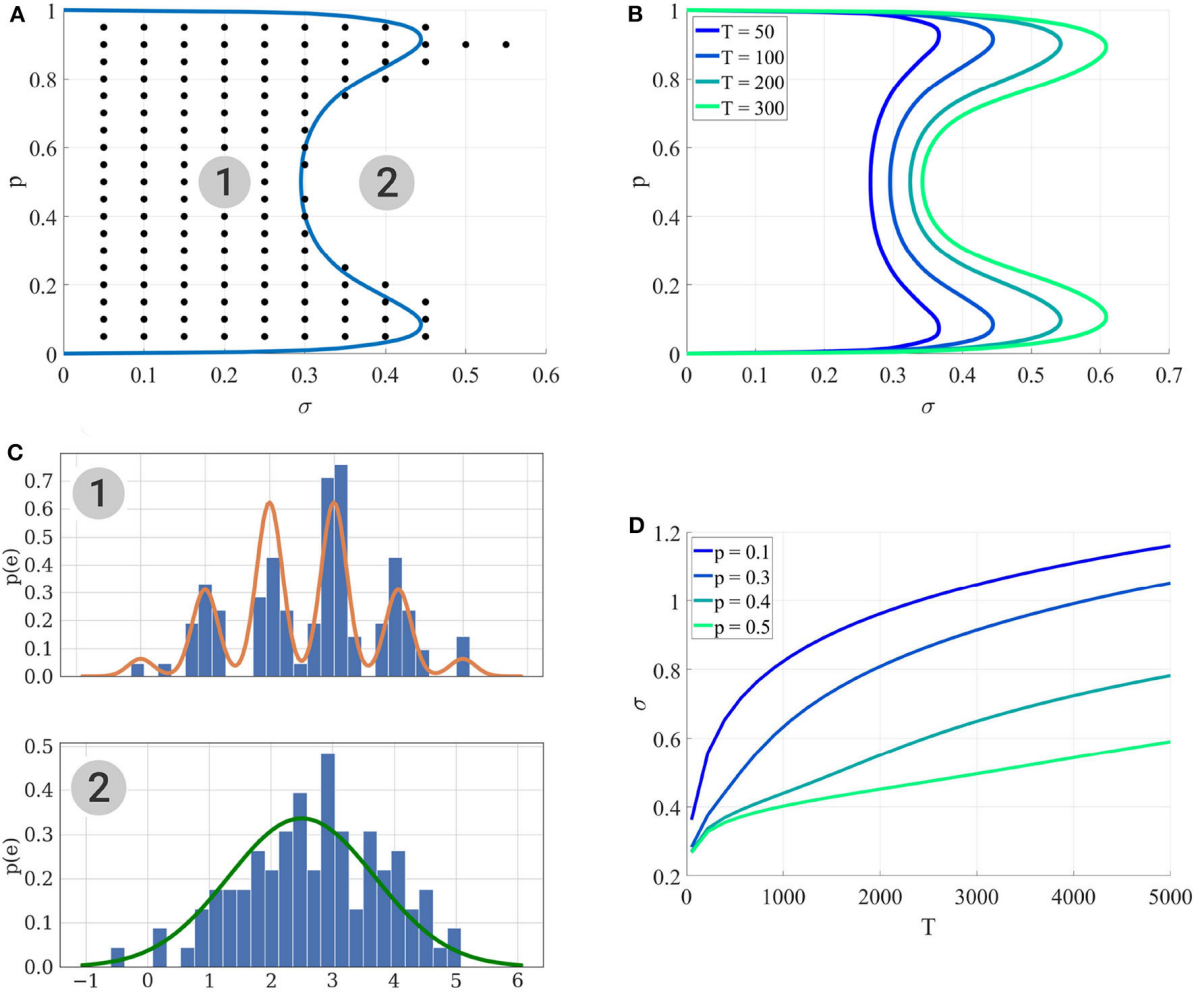
**(A)** Identifiability domain of $M_{1}$ as a function of *p* and σ. Blue line: domain of identifiability from Equation (18). On the left part of the blue line, the recording noise σ is sufficiently low to identify $M_{1}$. Black dots: values (p^*^, σ^*^) for which the average BIC of 400 data sets results in the correct identifiability of $M_{1}$. Results obtained for *N* = 5, *q* = 1, and *T* = 100. **(B)** Identifiability domain of the binomial model $M_{1}$ compared to a Gaussian distribution $M_{0}$, computed from (18), for different values of T. **(C)** To visualize the effect of σ on the data, this panel shows histograms of data generated from σ = 0.2 (1) and σ = 0.4 (2), alongside with their theoretical distribution from Equation (8) (orange line in the upper panel) or when a Gaussian distribution is fitted on them (green line in the lower panel). In the identifiability domain (1), quantal peaks are clearly visible. Outside of the identifiability domain (2), the binomial distribution becomes Gaussian-shaped. **(D)** Another visualization of the identifiability domains displayed in **(A,B)**. For different values of *p*, the maximum recording noise σ (i.e., the boundary of the identifiability domain) is plotted as a function of the number of data points *T*. The identifiability domain increases with *T*: intuitively, a larger data set facilitates the correct identification of a complex model.

*Per se*, the identifiability domain depends on all the parameters of $M_{1}$, as well as on the experimental protocol. For simplicity and in order to obtain a plot in 2 dimensions, we will only plot it as a function of *p* and σ while holding other variables to a fixed value. For a given experimental setup Ψ (which encompasses only the number of recorded data points *T*, the inter-spike intervals playing no role in these models), the following Markov-Chain-Monte-Carlo (MCMC) procedure is implemented:

1. A set of values p^*^ and σ^*^ are chosen from the space of possible values for *p* and σ;
2. Using p^*^ and σ^*^, 400 independent data sets ${\left(D_{i}\right)}_{1\leq i\leq 400}$ are generated from $M_{1}$. Each data set consists in *T* EPSCs;
3. For each $D_{i}$, the BIC of both models are computed; these values are averaged over *i* to compute an average BIC and identifiability is assessed if $M_{1}$ is preferred over $M_{0}$, which corresponds to the black dots in Figure 4A.

The procedure of plotting a complete identifiability domain can be quite time-consuming. Indeed, it requires to span the entire space of parameters; for each vector θ^*^, to generate a large number of independent data sets $\left(D_{i}\right)$; and for each of these data sets, to compute the maximum likelihood estimator $\hat{\theta }$ using the Expectation-Maximization algorithm (Barri et al., 2016). Details on the computation of $\hat{\theta }$ are available in Supplementary Material.

However, as both models $M_{0}$ and $M_{1}$ generate i.i.d. data, and by making the approximation $\hat{\theta }\approx \theta^{*}$ (i.e., by assuming that the maximum likelihood estimator $\hat{\theta }$ will be close to the true value θ^*^ from which data were generated), the condition that model $M_{1}$ is identifiable (17) can be approximated as follows:

(18) $$\begin{array}{l} -2T\int p\left(e|\theta^{*},M_{1}\right)\log p\left(e|\theta^{*},M_{1}\right)de+k_{M_{1}}\log\left(T\right)\leq \\ -2T\int p\left(e|\theta^{*},M_{1}\right)\log p\left(e|{\hat{\theta }}_{M_{0}},M_{0}\right)de+k_{M_{0}}\log\left(T\right) \end{array}$$

where ${\hat{\theta }}_{M_{0}}=\left(\mu ,\sigma^{2}\right)$ represents the mean μ ≈ N^*^p^*^q^*^ and the variance σ^2^ ≈ N^*^p^*^(1 − p^*^)q^*2^ + σ^*2^ of the data generated from $M_{1}$.

The condition specified by inequality (18) can be checked for any point θ^*^ without the need to generate a large number of independent data sets nor to compute the estimator $\hat{\theta }$. Solving (18) numerically for σ allows to draw the border of the identifiability domain of $M_{1}$, represented as solid lines in Figures 4A,B.

Several points are worth highlighting. Firstly, Figure 4A shows a good agreement between the results of the MCMC simulations (black dots) and those from the semi-analytical method (18) (blue line). Secondly, as expected, Figures 4A,B illustrate that the identifiability domain increases with the number of data points *T*: intuitively, a larger data set facilitates the correct identification of a complex model. Besides, irrespective of the values of *T* and σ, for *p* = 0 and *p* = 1 the model $M_{1}$ is structurally indistinguishable from a Gaussian distribution (see Figure 2A). Finally, the maximum noise σ which makes the binomial model $M_{1}$ indistinguishable from a Gaussian distribution $M_{0}$ is larger for extreme values of *p* (close to 0.9 or 0.1) than for *p* = 0.5. Indeed, in the latter case, the distributions of EPSC will be symmetric (as in the upper panel of Figure 4C), and hence just a little increase in recording noise will be enough to cover the inter-peak intervals and make the distribution Gaussian-shaped. In the former case, the distribution will be highly skewed, and thus difficult to approximate with a normal distribution.

The same approach can then be extended to more complicated models, by defining their identifiability domains as the part of the parameters plane where their average BIC will be lower than the BIC of a simpler one.

### 3.2. Identifiability Domain of the Binomial Model With Short Term Depression

**Figure 5:**
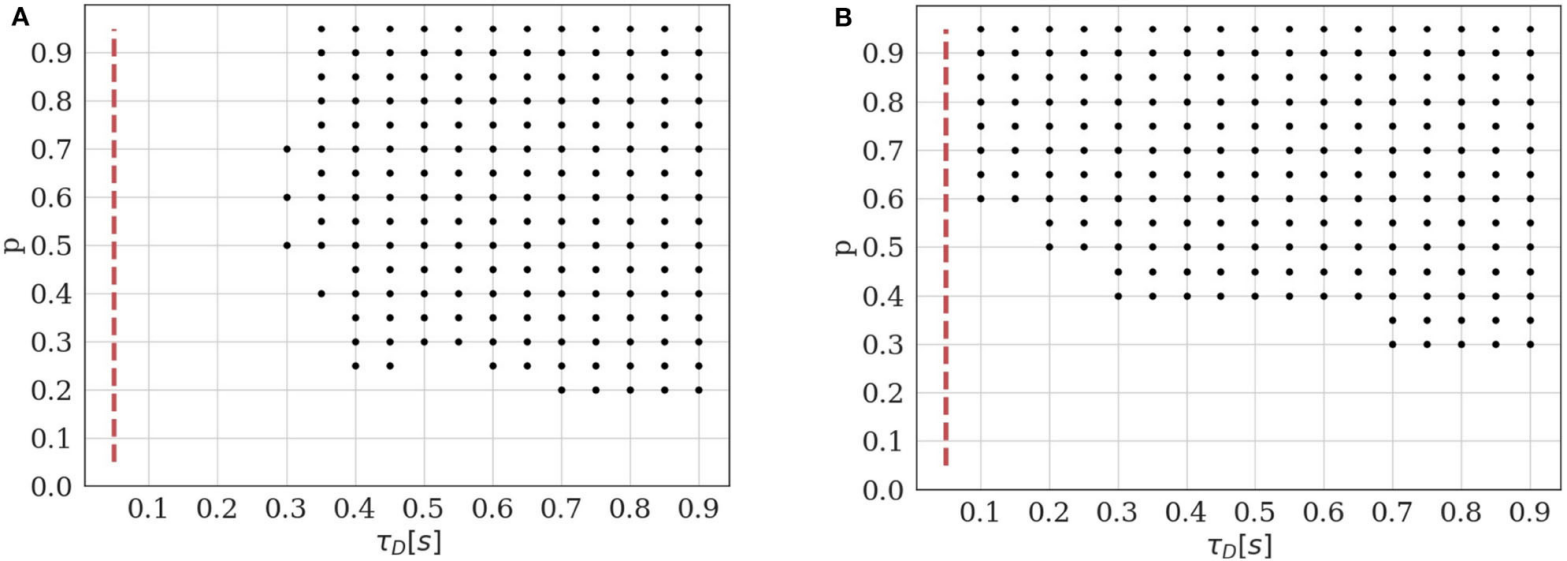
Identifiability domain of the binomial model with short-term depression $M_{2}$ as a function of *p* and τ_*D*_. Black dots correspond to the parameters for which the average BIC of 400 data sets results in the correct identifiability of the depressed model. Results obtained for *N* = 5, *q* = 1, σ = 0.2, and *T* = 100. **(A)** Constant stimulation protocol with an inter-spike interval *ISI* = 0.05*s* (red dotted line). **(B)** Stimulation protocol consisting in 20 repetitions of the same spike train: 4 spikes with an inter-spike interval *ISI* = 0.05*s* followed by a recovery spike 0.5*s* later.

We study here the conditions under which a binomial model with short-term depression ($M_{2}$) can be correctly identified from a model without short-term plasticity ($M_{1}$). In a first example, we assume that the presynaptic cell is stimulated at a constant inter-spike interval (ISI), which needs to be sufficiently short with respect to the time constant τ_*D*_ to make depression visible. We thus plot the identifiability domain as a function of both *p* and τ_*D*_. We use the same method as in 3.1: For each set of parameters p^*^ and $\tau_{D}^{*}$, 400 independent data sets are generated from $M_{2}$. Both models $M_{2}$ and $M_{1}$ are fitted on them, and black dots in Figure 5A correspond to the parameters for which the average BIC of $M_{2}$ is lower than the average BIC of $M_{1}$.

As expected, we verify that the identifiability of $M_{2}$ is only possible when τ_*D*_ is sufficiently long with respect to the inter-spike interval. Besides, if the release probability *p* is low, correlations between recordings will be weak and the effect of short-term depression will not be detectable. A major difference between models $M_{1}$ and $M_{2}$ is that, in the latter, observations {*e*_*i*_}_1 ≤ *i* ≤ *T*_ are not i.i.d. The value of the i-th recorded EPSC is a function of the number of available and released vesicles *n*_*i*_ and *k*_*i*_, which in turn depend on their previous values and on the ISI Δ*t*_*i*_. This has two main consequences. Firstly, using the same approximation as in (18) would lead to a biased estimate of the identifiability domain. Secondly, the classical definition of the BIC (15) should not be used since observations are correlated. Rather, we use Equation (16) to compare the evidence for $M_{1}$ and $M_{2}$ for a given data set.

Plotting the identifiability domain of a model also allows to investigate how the identifiability depends on the experimental protocol. For model $M_{1}$, we already saw that the identifiability domain increases with the number of data points *T* (see Figures 4B,D): a larger data set is more informative and allows for more reliable inference. In this case, *T* is the only experimental variable, as observations {*e*_*i*_}_1 ≤ *i* ≤ *T*_ are i.i.d. On the other hand, the identifiability domain of $M_{2}$ will depend not only on the number of data points, but also on the stimulation protocol. We compare the constant stimulation protocol (*T* data points with a constant inter-spike interval *ISI* = 0.05*s*) of Figure 5A with a more realistic stimulation protocol in Figure 5B. In electrophysiological recordings, synaptic transmission is classically studied by stimulating the presynaptic cell with short regular train of spikes at a given frequency, followed by a recovery spike. This protocol is then repeated several times (Costa et al., 2013; Barri et al., 2016; Bird et al., 2016). Such periodic trains are more informative than a constant stimulation protocol, as they allow to probe a broader range of temporal dynamics.

In Figure 5B, we use 20 repetitions of a train of 4 spikes at 20Hz (*ISI* = 0.05*s*), followed by a recovery spike 0.5*s* later. This protocol entails the same number of data points *T* = 100 as the constant one, but allows to identify STD for a broader range of depression time constants (namely, for τ_*D*_ < 0.3*s*). On the other hand, since there are fewer successive stimulations within a short time interval than in the constant protocol, depression can only be identified when the release probability *p* is sufficiently high to induce vesicle pool depletion.

### 3.3. Data Free Model Selection

In model-based inference of synaptic parameters, a crucial step related to the estimation of the parameters is model selection, which is usually performed in several steps:

1. Data D are acquired from a synapse using protocol Ψ;
2. A nested family of *n* + 1 possible models $F=\left\{M_{0},M_{1},...,M_{n}\right\}$ is defined;
3. Each of these models is fitted on D to obtain *n* + 1 MLE ${\hat{\theta }}_{0},{\hat{\theta }}_{1},...,{\hat{\theta }}_{n}$;
4. A model selection criterion (Bayes Factor, BIC, AIC.) is computed to quantify and rank the fitness of each model on D;
5. If $M_{i}$ is the selected model, then its MLE ${\hat{\theta }}_{i}$ is selected as the inference of synaptic parameters.

However, in many studies (Barri et al., 2016; Bird et al., 2016; Ghanbari et al., 2017), such a model selection step is not described. In this section, we investigate the possibility, having only access to the inferred values $\hat{\theta }$ of the parameters and to the description of the experimental protocol Ψ, to verify that the model used to infer $\hat{\theta }$ was indeed practically identifiable (i.e., to verify if a simpler model would have given a better fit to the data).

We use the notation D for a set of data generated from a model M parameterized with θ^*^, and $\hat{\theta }$ the inferred parameters obtained by fitting the parameters θ of model M on D. If θ^*^ is within the practical identifiability domain of M as we defined it, it is then possible to correctly infer it from D, and hence $\hat{\theta }\approx \theta^{*}$ will also be within the identifiability domain of M. Reciprocally, if $\hat{\theta }$ is not in the identifiability domain of M, then a submodel would have provided a better fit to the data D than M. Is it thus possible to verify if M overfits the data simply by verifying if $\hat{\theta }$ is in its identifiability domain, without having access to the data.

**Figure 6:**
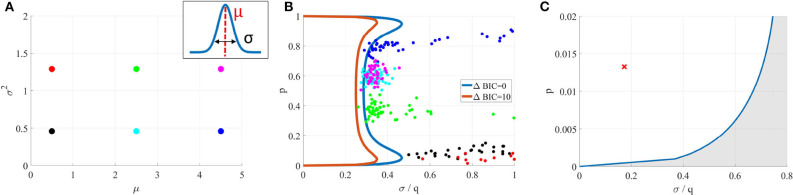
**(A)** Data sets were generated from a Gaussian model $M_{0}$ (for six different means μ and variances σ^2^). **(B)** A binomial model $M_{1}$ was fitted on them. In each case, inferred parameters (colored dots) are out of the identifiability domain of $M_{1}$. It is thus possible to verify if a model used to fit data was indeed identifiable, without having access to the data and only using inferred parameters. **(C)** Blue line: identifiability domain of the binomial model compared to a Gaussian distribution, for *N* = 42 and *T* = 328, computed from (18). The red cross corresponds to the parameters inferred from (Del Castillo and Katz, 1954), and is indeed within the identifiability domain.

This is illustrated in Figures 6A,B, where $M_{1}$ is fitted on data generated from its submodel $M_{0}$. For six different values of $\theta_{0}^{*}$ (Figure 6A), the inferred parameters are out of the identifiability domain of $M_{1}$ (Figure 6B), showing that data are indeed better explained by $M_{0}$ than by $M_{1}$.

#### 3.3.1. First Example: Application to the Data From Katz et al. (1954)

We first apply our *data free* model selection method to the seminal 1954 paper from Del Castillo and Katz (1954), in which the quantal nature of neurotransmitter release is identified for the first time. In order to observe mEPSP, they artificially reduced the release probability *p* by lowering the external calcium concentration. Although the quantal components of postsynaptic potentials are clearly visible and thoroughly analyzed, it would be interesting to verify, using our proposed model identifiability analysis method, that the binomial model (i.e., a multi-quantal distribution) indeed provides a better fit to the data than a simpler Gaussian model (i.e., a uni-quantal distribution).

Data (Fatt and Katz, 1952) consist in 328 EPSPs recorded at the neuro-muscular junction (NMJ) of a frog muscle. Fitting the binomial model and running the Expectation-Maximization algorithm on them yields $\hat{N}=42$, $\hat{p}=0.013$, $\hat{q}=0.875\;mV$, and $\hat{\sigma }=0.15\;mV$ (and hence $\frac{\hat{\sigma }}{\hat{q}}\approx 17\%$). For this particular example, we have not only access to the inferred parameters $\hat{\theta }$, but also to the data: it is thus possible to directly compare the BIC of a Gaussian (${\text{BIC}}_{M_{0}}=764.95$) and of a binomial (${\text{BIC}}_{M_{1}}=470.37$) distributions, which indeed confirms that data are better explained by the binomial quantal model.

However, even without the data, we can verify that the point in the parameter-protocol space specified by $\hat{\theta }$ (the inferred values of the parameters) and Ψ (the number of data points *T* = 328) is indeed within the identifiability domain of the binomial model $M_{1}$ compared to $M_{0}$ (see Figure 6C), thus confirming the multi-quantal nature of the recordings.

#### 3.3.2. Second Example: Application to the Data From Barri et al. (2016)

We then apply our method to the results presented in the 2016 paper from Barri et al. (2016), in which the complete binomial model (with STD and STF) is fitted on recordings from layer 5 pyramidal neurons. They use a slight variation of the binomial release model with short term plasticity described by Equations (1)–(6), in which the emission probability does not follow a Gaussian, but an inverse Gaussian distribution:

(19) $$\begin{array}{l} p_{\theta }\left(e_{i}|k_{i}\right)=\frac{q^{3/2}k_{i}}{\sqrt{2\pi \sigma^{2}e_{i}^{3}}}\exp\left(-\frac{q{\left(e_{i}-qk_{i}\right)}^{2}}{2\sigma^{2}e_{i}}\right) \end{array}$$

To verify that the data would not have been better fitted by a simpler model (and hence, that the published estimates of synaptic parameters are reliable), 100 synthetic data sets were generated from the complete binomial model using the stimulation protocol and the inferred values of the parameters described in (Barri et al., 2016):

- 20 repetitions of the same stimulation protocol consisting in 8 presynaptic spikes at 20Hz followed by a recovery spike 500 ms later;
- N^*^ = 17, p^*^ = 0.27, q^*^ = 0.18 *mV*, σ^*^ = 0.06 *mV*, $\tau_{D}^{*}=202\;ms$, and $\tau_{F}^{*}=449\;ms$.

**Figure 7:**
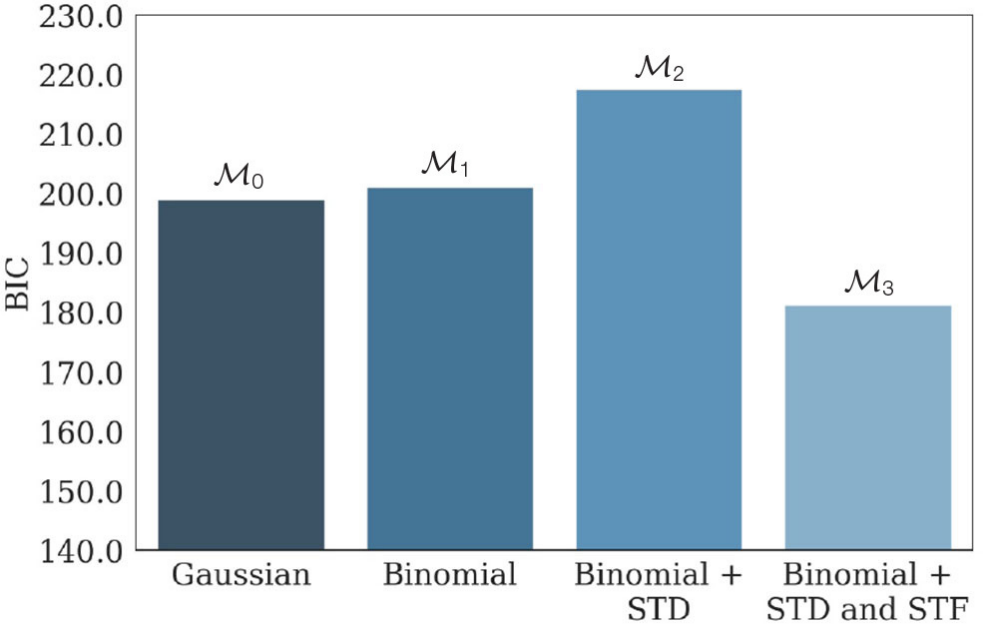
Average BIC of $M_{0}$, $M_{1}$, $M_{2}$, and $M_{3}$ when fitted on 100 independent data sets generated from $M_{3}$ parameterized with N^*^ = 17, p^*^ = 0.27, q^*^ = 0.18 *mV*, σ^*^ = 0.06 *mV*, $\tau_{D}^{*}=202\;ms$, and $\tau_{F}^{*}=449\;ms$. $M_{3}$ has the lowest average BIC compared to its submodels, showing that the parameters used to generate the data are indeed within the identifiability domain of $M_{3}$. As a consequence, we can infer that $M_{3}$ indeed provided the best fit to the data compared to its submodels, and that inferred parameters presented in Barri et al. (2016) are reliable. The facilitating nature of the synapse is illustrated by the fact that the BIC of $M_{2}$ (the model with only STD and no STF) is substantially larger than the one of $M_{3}$.

$M_{0}$, $M_{1}$, $M_{2}$, and $M_{3}$ were then fitted on the generated data. Average values of their respective BIC are presented in Figure 7, and confirm the identifiability of the model used in the study.

## 4. Discussion

Obtaining an accurate estimate of the parameters of a system from noisy and scarce observations is a crucial problem in neuroscience. Especially, different methods have been proposed for estimating the parameters describing a synapse (namely, its number of independent release sites, their release probability upon the arrival of a presynaptic spike, the quantum of current elicited by one release event, the time constants of depression and facilitation, etc.). Inferring their values allows to analyze the locus of synaptic plasticity and homeostasis; to study possibly synapse-related diseases; and more generally to investigate learning, memory, and neural dynamics, which are mediated by synaptic transmission.

It is usually impossible to measure directly these parameters. However, they can be estimated by fitting a biophysical model of synapse on currents recorded on the post-synaptic side and elicited by experimental stimulation of the presynaptic cell. This approach for estimating the parameters of a system is referred to as model-based inference. As different competing models may be used to describe the system and explain its output, model-based inference of parameters thus raises the question of what makes a good model.

Prior to any data recording, a required property for competing models is identifiability. Although structural identifiability has been widely studied, no quantitative criterion exists for practical identifiability, which is usually only qualitatively assessed. Here, we propose a definition for the practical identifiability of a model, based on its expected support given the distribution of the data. We define the practical identifiability domain of a statistical model as the set of parameters for which the model is correctly identified as the ground truth compared to a simpler alternative submodel, and we study the identifiability domains of different models of synaptic release. In the process, we propose an extension of the Bayesian Information Criterion (BIC) for models with correlated data. The BIC is a widely used tool for model selection, but it is derived by assuming that the outputs of the system are mutually independent, which is not the case for models of chemical synapse. Finally, we show that our approach allows to perform *data free* model selection, i.e., to verify the identifiability of a model without having access to the data.

The definition of practical identifiability we introduced here differs from the influential contribution of Raue et al. (2009) in two ways. Firstly, our definition is data-independent: it does not only hold for a specific set of recorded data D. Indeed, we define practical identifiability as an intrinsic property of the model M and experimental protocol Ψ. We actually define the *a priori* expected identifiability before a specific D is recorded, which allows to study how identifiability is affected by different experimental protocols. Secondly, since our definition is derived from a model-selection argument, it does not require to select a possibly arbitrary threshold on the practical identifiability of parameters. Rather, it is defined with respect to a particular submodel. Although the choice of the submodel might itself be arbitrary, we argue that nested models and families naturally arise in commonly used statistical techniques, such as polynomial regression (Edwards and MacCallum, 2012), or Generalized Linear Models (GLM) (Pillow et al., 2008). Especially, the widespread use of phenomenological models in neuroscience (Kobayashi et al., 2009; Melanson et al., 2014; Wang et al., 2016; Levenstein et al., 2020) makes the use of nested families and submodels relevant.

Another limitation of our approach is its practical implementation. As mentioned, the model evidence p(D|M), on which our definition is based, is often intractable in practice for complex models, and needs to be estimated. For practical purpose, we used the Bayesian Information Criterion (BIC) to compute the identifiability domains of our different models of synapse. However, we acknowledge that the BIC only provides a valid approximation of the model evidence when the number of samples is sufficiently large. A future step would be to study the robustness of our approach to different computations of the model evidence or to other approximations, such as the Akaike Information Criterion (AIC) (Burnham and Anderson, 2004).

Our identifiability domains are similar to the approach adopted in Koyama (2012), in which the authors study under which regime of rate fluctuation are the temporal variations of a neuron firing rate correctly identified. Spike trains are generated from a model of spiking neuron with a fluctuating firing rate (complex model); but under a certain value of rate fluctuation, this model becomes indistinguishable from a model of spiking neuron with a constant rate (simple model). Plotting the identifiability of the fluctuating-rate model as a function of the amplitude of rate fluctuation allows them to identify which distribution of inter-spike intervals has the broader identifiability domain (and thus maximizes the efficiency of rate fluctuation transmission).

In model-based inference and parameter estimation, one is often interested in obtaining theoretical bounds on the achievable error performance. Such theoretical bounds allow to assess *a priori* the possibility to correctly infer the parameters. A well-known theoretical result is the Cramér-Rao bound (Van Trees, 2004; Van Trees and Bell, 2007), which provides a lower bound on the variance of the parameter estimator. This bound, which depends on the model, its parameters, and the experimental protocol, may actually be too loose in practice, and does not account for the threshold effect described in Kostal et al. (2015). In many cases, as the number of data points increases, the estimate error displays a threshold-like transition, from a region of low performance to a region of high performance where the Cramér-Rao bound is attained. Our definition of practical identifiability also discriminates between regions of low information (for small signal-to-noise ratios and sample size) and high accuracy, provides a quantitative criterion to discriminate them, and can be extended to the case of non-i.i.d. data. An interesting future step would be to verify how the boundaries of our proposed identifiability domains compare with the transition threshold described in Kostal et al. (2015).

An interesting topic would be to study the practical identifiability domain as the number of observations *T* goes to infinity. In this asymptotic case, practical non-identifiability means that the model cannot be identified, even with an infinite amount of data. We can conjecture that practical identifiability is equivalent to structural identifiability in this asymptotic case, as hinted by Figure 4: the identifiability domain increases with *T*. A future step would be to verify if the practical identifiability domain of a model is included in its structural identifiability domain, and how it behaves when the number of observations *T* goes to infinity.

We applied our analysis to four variants of the binomial model, of increasing complexity: a Gaussian model (i.e., a uni-quantal distribution); a binomial model without short-term plasticity; a binomial model with only short-term depression; and a binomial model with both short-term depression and facilitation. A future step would be to extend our analysis to further generalizations of the binomial model, in order to account for parameters heterogeneity. Especially, the binomial model assumes that the release probability and the quantal amplitude are identical for each release site. It is however possible to hypothesize that there are several pools of vesicles, each having different parameters (for instance a fast depleting pool and a slow depleting pool). There will be regimes in which those sub-pools can be detected and other in which the noise is too high or the experimental protocol not informative enough to identify them, which can be quantified using our definition of identifiability. Another possible generalization of the binomial model is to assume that the postsynaptic response to one vesicle release is not fixed, but follows for instance a Gamma distribution (Bhumbra and Beato, 2013) to account for variability in vesicles size and neurotransmitter content.

Model selection is not only a first step in model-based inference of synaptic parameters (as it is necessary to have a reliable estimates of the parameters), but also a tool to study the mechanisms of neurotransmitter release at a chemical synapse. An alternative hypothesis (e.g., “*this synapse shows short-term plasticity”*) can be compared to a null hypothesis (“*this synapse does not show short-term plasticity”*) by computing how well the complex model (i.e. with short-term plasticity) explains the behavior of the synapse compared to the simple model (i.e., without short-term plasticity). Testing models of growing complexity allows to study the nature of the synapse and to identify mechanisms of neurotransmitter release. But the possibility to correctly select the model that corresponds to the true behavior of the synapse will depend on its parameters and on the experimental protocol used to record data: there are regimes in which the specific features of a model do not appear in the data. Such regimes correspond to the identifiability domain of the model, and studying them allows to draw conclusions on the nature of the synapse.

As stated previously, the problem of inferring parameters from noisy and scarce observations is not restricted to synaptic parameters estimation, but is a crucial question in neuroscience. Our proposed methodology could also be applied to models of single neurons (Koch, 2004; Jolivet et al., 2008; Gerstner and Naud, 2009; Mensi et al., 2012), neural population dynamics (René et al., 2020), or calcium-driven vesicles fusion (Schneggenburger and Neher, 2000; Lou et al., 2005; Sun et al., 2007).

On a broader scale, instead of seeing parametric non-identifiability as a statistical problem, we could consider it as a biophysical feature. The total synaptic strength between two cells is a function of both presynaptic (*N, p*) and postsynaptic (*q*) parameters. Different combinations of these parameters could lead to the same average postsynaptic response: a presynaptic modification of the number of release sites *N* can be compensated by an inverse modification of the postsynaptic number of receptors affecting *q*. This combined effect of presynaptic and postsynaptic plasticity has been shown to enable reliable and flexible learning (Costa et al., 2015) and homeostatic modulation (Davis and Müller, 2015). More generally, the question of degeneracy, defined as the ability of different elements to perform the same function, could be addressed within the framework of identifiability analysis (Drion et al., 2015; Rathour and Narayanan, 2019).

Finally, our proposed definition of model identifiability is paving the way toward Optimal Experiment Design (OED) for model selection and parameter inference. The information conveyed by the data about the ground truth model and its parameters depends on the experimental protocol: number of recorded data points, stimulation frequency, etc. The goal of OED is to optimize the experimental protocol in order to maximize the possibility to discriminate between competing models (Vanlier et al., 2014; Balietti et al., 2018) and the precision of the inference of their parameters. An OED for inferring the parameters of a given model maximizes the mutual information between the data and the parameters I(D,θ) (Huan and Marzouk, 2013). This quantity turns out to be equal to the expected gain in information about θ (defined as the Kullback-Leibler divergence between its prior and its posterior), on which our proposed definition of the informative domain (11) is based. Similarly, maximizing the Average Log Bayes Factor (13) is equivalent to maximizing the discriminability between the two models M and $M^{\prime }$, and hence finding an OED for model selection. As a thorough theoretical preliminary analysis of the properties of the competing models is a first step prior to model selection and parameter inference (Asprey and Macchietto, 2000), we believe that our theoretical contribution to model analysis will contribute to the development of OED techniques for synaptic transmission study.

## Data Availability Statement

Matlab and Python files are available in the following GitHub repository: https://github.com/camillegontier/identifiability_binomial.git.

## Author Contributions

CG and J-PP wrote the paper and derived the equations. CG performed the numerical simulations, made the figures, and wrote the MATLAB code and the Python code. J-PP conceived of the presented idea and supervised the project. All authors contributed to the article and approved the submitted version.

## Conflict of Interest

The authors declare that the research was conducted in the absence of any commercial or financial relationships that could be construed as a potential conflict of interest.

## Footnotes

^1^It is also possible to perform model-based inference of synaptic parameters based on post-synaptic spike trains instead of EPSCs, as in Ghanbari et al. (2017, 2020)

^2^The outputs of a model of chemical synapse can be either electric postsynaptic currents (EPSC) or potentials (EPSP). In the latter case, σ and *q* will be expressed in [V] instead of [A].

^3^Other distributions can also be used for the emission probability. Barri et al. (2016) assumed an inverse Gaussian to account for the observed right-skewness of mEPSP (Bekkers et al., 1990; Bhumbra and Beato, 2013)
