# Supplementary material for: Identifiability of a Binomial Synapse
Source: Front Comput Neurosci. 2020 Sep 30;14:558477. doi: 10.3389/fncom.2020.558477 (PMC7561371; doi:10.3389/fncom.2020.558477)
Supplement: Supplementary file 1 [file Data_Sheet_1.PDF]

Supplementary Material

We use the following notations:

- $\mathbf{n} = \{n_i\}_{1 \leq i \leq T}$, $\mathbf{k} = \{k_i\}_{1 \leq i \leq T}$, and $\mathcal{D} = \{e_i\}_{1 \leq i \leq T}$ represent respectively the number of available vesicles at the moment of spike i, the number of vesicles released after spike i (hidden states), and the i-th recorded EPSC (observations);
- θ_l and θ_m are the l-th and m-th elements of the vector θ;
- For simplicity, we use the shorthand notation $p_\theta(\mathcal{D}) = p(\mathcal{D}|\theta, \mathcal{M})$.

1 DERIVATION OF THE BIC

We define $Q(\theta) = \log p(\mathcal{D}|\theta, \mathcal{M})$, which can be approximated using a second-order Taylor series around the MLE $\hat{\theta}$:

$$Q(\theta) \approx Q(\hat{\theta}) + \frac{1}{2}(\theta - \hat{\theta})^T H(\hat{\theta})(\theta - \hat{\theta}) \tag{S1}$$

where $H(\hat{\theta})$ is the Hessian matrix of Q expressed at $\hat{\theta}$. Using (S1) in $p(\mathcal{D}|\mathcal{M}) = \int_\theta p(\mathcal{D}|\theta, \mathcal{M})\pi(\theta)d\theta$, and assuming a flat prior for θ given $\mathcal{M}$ yields

$$p(\mathcal{D}|\mathcal{M}) \approx \exp(Q(\hat{\theta}))\pi(\hat{\theta}) \int_\theta \exp\left(\frac{1}{2}(\theta - \hat{\theta})^T H(\hat{\theta})(\theta - \hat{\theta})\right) d\theta$$

Since $H(\hat{\theta})$ is symmetric and negative-definite, the integral can be computed using Laplace's method:

$$\log p(\mathcal{D}|\mathcal{M}) \approx \log p(\mathcal{D}|\hat{\theta}, \mathcal{M}) + \log \pi(\hat{\theta}) + \frac{k_\mathcal{M}}{2}\log(2\pi) - \frac{1}{2}\log(|-H(\hat{\theta})|) \tag{S2}$$

Assuming that the number T of data points is sufficiently large, and that data in $\mathcal{D}$ are independent and identically distributed (IID), the following simplification can be made:

$$[H(\hat{\theta})]_{l,m} = \frac{\partial^2}{\partial\theta_l \partial\theta_m} \log p(\mathcal{D}|\theta, \mathcal{M})\Big|_{\hat{\theta}} = \frac{\partial^2}{\partial\theta_l \partial\theta_m} \sum_{i=1}^{T} \log p(e_i|\theta, \mathcal{M})\Big|_{\hat{\theta}} = T\frac{1}{T}\sum_{i=1}^{T} \frac{\partial^2}{\partial\theta_l \partial\theta_m} \log p(e_i|\theta, \mathcal{M})\Big|_{\hat{\theta}}$$

$$\implies [H(\hat{\theta})]_{l,m} \approx -T\mathcal{I}(\hat{\theta})$$

where $\mathcal{I}(\hat{\theta})$ is the empirical Fisher Information Matrix for a single observation expressed at $\hat{\theta}$. (S2) thus becomes

$$\log p(\mathcal{D}|\mathcal{M}) \approx \log p(\mathcal{D}|\hat{\theta}, \mathcal{M}) + \log \pi(\hat{\theta}) + \frac{k_{\mathcal{M}}}{2}\log(2\pi) - \frac{k_{\mathcal{M}}}{2}\log(T) - \frac{1}{2}\log(|\mathcal{I}(\hat{\theta})|)$$

Finally, ignoring the terms that do not depend on T (for T sufficiently large) and multiplying both sides of the equality by -2 leads to $BIC_{\mathcal{M}}(\mathcal{D}) \approx -2\log p(\mathcal{D}|\mathcal{M})$.

2 INFERRING THE VALUES OF SYNAPTIC PARAMETERS USING THE EXPECTATION-MAXIMIZATION ALGORITHM

We define the quantity Q as

$$Q(\theta|\theta^t) = \langle \log p_\theta(\mathcal{D}, \mathbf{n}, \mathbf{k}) \rangle_{p_{\theta^t}(\mathbf{n},\mathbf{k}|\mathcal{D})}$$

that is to say the expected value of the log likelihood function of θ $\log p_\theta(\mathcal{D}, \mathbf{n}, \mathbf{k})$ with respect to the conditional distribution of the hidden variables **n** and **k** given the observations $\mathcal{D}$ and the current estimates of the parameters θ^t. The M-step of the algorithm corresponds to the maximization of this quantity with respect to θ:

$$\theta^{t+1} = \arg\max_\theta Q(\theta|\theta^t)$$

which can be realized by computing the null-point of its gradient with respect to θ:

$$\langle \nabla_\theta \log p_\theta(\mathcal{D}, \mathbf{n}, \mathbf{k}) \rangle_{p_{\theta^t}(\mathbf{n},\mathbf{k}|\mathcal{D})} \Big|_{\theta^{t+1}} = 0 \tag{S3}$$

The derivative of the joint probability with respect to each of the parameters can be analytically computed and then introduced into (S3). Whenever possible, we use an analytical solution for θ^{t+1}, in order to accelerate each iteration of the algorithm. For any quantity $f(n_i, k_i)$, the average can be conveniently computed by using the marginal distributions of vectors **n** and **k** that share the same values at time i n_i and k_i:

$$\langle f(n_i, k_i) \rangle_{p_{\Theta^t}(\mathbf{n},\mathbf{k}|\mathcal{D})} = \sum_{n_i,k_i} f(n_i, k_i) p_{\Theta^t}(n_i, k_i|\mathcal{D})$$

Similarly, marginal distributions can be computed for any temporal indexes. For instance:

$$\langle n_i - n_{i-1} + k_{i-1} \rangle_{p_{\Theta^t}(\mathbf{n},\mathbf{k}|\mathcal{D})} = \sum_{n_i,n_{i-1},k_{i-1}} (n_i - n_{i-1} + k_{i-1}) p_{\Theta^t}(n_i, n_{i-1}, k_{i-1}|\mathcal{D})$$

The probability $p_{\Theta^t}(n_i, k_i|\mathcal{D})$ can then be computed using the Baum-Welch algorithm. As $(\log(u))' = \frac{u'}{u}$, we obtain the following equations for $\mathcal{M}_1$, $\mathcal{M}_2$, and $\mathcal{M}_3$ respectively:

2.1 Binomial model without STP

M-step equations for updating the parameters p, q, and σ of model $\mathcal{M}_1$ (corresponding to the roots of Eq. (S3)):

$$q^{t+1} = \frac{\sum_{i=1}^{T} \langle e_i k_i \rangle}{\sum_{i=1}^{T} \langle k_i^2 \rangle}$$

$$\sigma^{t+1} = \sqrt{\frac{\sum_{i=1}^{T} \langle (e_i - q^{t+1} k_i)^2 \rangle}{T}}$$

$$p^{t+1} = \frac{\sum_{i=1}^{T} \langle k_i \rangle}{NT}$$

2.2 Binomial model with STD

M-step equations for updating the parameters p, q, σ, and τ_D of model $\mathcal{M}_2$ (corresponding to the roots of Eq. (S3)):

$$q^{t+1} = \frac{\sum_{i=1}^{T} \langle e_i k_i \rangle}{\sum_{i=1}^{T} \langle k_i^2 \rangle}$$

$$\sigma^{t+1} = \sqrt{\frac{\sum_{i=1}^{T} \langle (e_i - q^{t+1} k_i)^2 \rangle}{T}}$$

$$\frac{dQ(\Theta|\Theta^t)}{d\tau_D} = 0 \implies \sum_{i=2}^{T} \frac{\partial I_i}{\partial \tau_D} \left\langle \frac{n_i - n_{i-1} + k_{i-1}}{I_i} - \frac{N - n_i}{1 - I_i} \right\rangle = 0$$

(the sum starts at $i = 2$ since τ_D does not appear in the probability of the first EPSC) with $I_i = 1 - \exp\left(-\frac{\Delta t_i}{\tau_D}\right)$

$$p^{t+1} = \frac{\sum_{i=1}^{T} \langle k_i \rangle}{\sum_{i=1}^{T} \langle n_i \rangle}$$

2.3 Binomial model with STD and STF

M-step equations for updating the parameters p, q, σ, τ_D, and τ_F of model $\mathcal{M}_3$ (corresponding to the roots of Eq. (S3)):

$$q^{t+1} = \frac{\sum_{i=1}^{T} \langle e_i k_i \rangle}{\sum_{i=1}^{T} \langle k_i^2 \rangle}$$

$$\sigma^{t+1} = \sqrt{\frac{\sum_{i=1}^{T} \langle (e_i - q^{t+1} k_i)^2 \rangle}{T}}$$

$$\frac{dQ(\Theta|\Theta^t)}{dp} = 0 \implies \sum_{i=1}^{T} \left\langle \frac{\partial u_i}{\partial p} \left(\frac{k_i}{u_i} - \frac{n_i - k_i}{1 - u_i} \right) \right\rangle = 0$$

with

$$\frac{\partial u_i}{\partial p} = 1 + \frac{\partial u_{i-1}}{\partial p}(1-p)\exp\left(-\frac{\Delta t_i}{\tau_F}\right) - u_{i-1}\exp\left(-\frac{\Delta t_i}{\tau_F}\right)$$

and $\frac{\partial u_1}{\partial p} = 1$

$$\frac{dQ(\Theta|\Theta^t)}{d\tau_F} = 0 \implies \sum_{i=1}^{T}\left\langle \frac{\partial u_i}{\partial \tau_F}\left(\frac{k_i}{u_i} - \frac{n_i - k_i}{1-u_i}\right)\right\rangle = 0$$

with

$$\frac{\partial u_i}{\partial \tau_F} = (1-p)\left(\frac{\partial u_{i-1}}{\partial \tau_F}\exp\left(-\frac{\Delta t_i}{\tau_F}\right) + u_{i-1}\exp\left(-\frac{\Delta t_i}{\tau_F}\right)\frac{\Delta t_i}{\tau_F^2}\right)$$

and $\frac{\partial u_1}{\partial \tau_F} = 0$

$$\frac{dQ(\Theta|\Theta^t)}{d\tau_D} = 0 \implies \sum_{i=2}^{T}\frac{\partial I_i}{\partial \tau_D}\left\langle \frac{n_i - n_{i-1} + k_{i-1}}{I_i} - \frac{N - n_i}{1 - I_i}\right\rangle = 0$$

The EM algorithm is run for different possible values of N; for each of these varying values, a MLE $\hat{\theta}_N$ is computed for the continuous parameters. The value of $\hat{\theta}_N$ yielding the highest likelihood is the global MLE. To minimize the vulnerability to local minima, each run of the EM algorithm needs to be repeated with different initial values θ^0 for the continuous parameters. However, to minimize computation time while plotting the identifiability domain of $\mathcal{M}_2$, the algorithm was only run once with the parameters (including N) initiated at their ground-truth values (i.e. $\theta^0 = \theta^*$).

3 BAUM-WELCH ALGORITHM AND COMPUTATION OF THE HESSIAN MATRIX

The elements of the Hessian matrix $H(\theta)$ are the second derivatives of the log-likelihood

$$[H(\theta)]_{l,m} = \frac{\partial^2}{\partial\theta_l \partial\theta_m}\log p_\theta(\mathcal{D})$$

We define the following elements:

– The transition matrix A:

$$A(n', k', n, k, i) = p_\theta(n_i = n, k_i = k | n_{i-1} = n', k_{i-1} = k')$$

– The initial state distribution:

$$\pi_{n,k} = p_\theta(n_1 = n, k_1 = k)$$

– The observation matrix:

$$b_{n,k}(i) = p_\theta(e_i | n_i = n, k_i = k)$$

– The forward probability $\alpha_{n,k}(i) = p_\theta(e_1, \ldots, e_i, n_i = n, k_i = k)$ which is defined recursively:
  1. $\alpha_{n,k}(1) = \pi_{n,k} b_{n,k}(1)$

2. $\alpha_{n,k}(i+1) = b_{n,k}(i+1)\sum_{n',k'}\alpha_{n',k'}(i)A(n',k',n,k,i+1)$

Finally:

$$p_\theta(\mathcal{D}) = \sum_{n,k}\alpha_{n,k}(T)$$

$[H(\theta)]_{l,m}$ is computed as follows:

$$\frac{\partial^2}{\partial\theta_l\theta_m}\log p_\theta(\mathcal{D}) = -\frac{1}{p_\theta(\mathcal{D})^2}\frac{\partial p_\theta(\mathcal{D})}{\partial\theta_l}\frac{\partial p_\theta(\mathcal{D})}{\partial\theta_m} + \frac{1}{p_\theta(\mathcal{D})}\sum_{n,k}\frac{\partial^2}{\partial\theta_l\theta_m}\alpha_{n,k}(T)$$

with

$$\frac{\partial p_\theta(\mathcal{D})}{\partial\theta_l} = \sum_{n,k}\frac{\partial\alpha_{n,k}(T)}{\partial\theta_l}$$

and where the derivatives of the α parameters from the Baum-Welch algorithm are computed recursively:

$$\frac{\partial\alpha_{n,k}(i+1)}{\partial\theta_l} = \frac{\partial b_{n,k}(i+1)}{\partial\theta_l}\sum_{n',k'}\alpha_{n',k'}(i)A(n',k',n,k,i+1)$$
$$+b_{n,k}(i+1)\sum_{n',k'}\left(\frac{\partial\alpha_{n',k'}(i)}{\partial\theta_l}A(n',k',n,k,i+1) + \alpha_{n',k'}(i)\frac{\partial A(n',k',n,k,i+1)}{\partial\theta_l}\right)$$

and

$$\frac{\partial^2}{\partial\theta_l\theta_m}\alpha_{n,k}(i+1) = \frac{\partial^2}{\partial\theta_l\theta_m}b_{n,k}(i+1)\sum_{n',k'}\alpha_{n',k'}(i)A(n',k',n,k,i+1)$$
$$+\frac{\partial b_{n,k}(i+1)}{\partial\theta_l}\sum_{n',k'}\left[\frac{\partial\alpha_{n',k'}(i)}{\theta_m}A(n',k',n,k,i+1) + \alpha_{n',k'}(i)\frac{\partial A(n',k',n,k,i+1)}{\theta_m}\right]$$
$$+\frac{\partial b_{n,k}(i+1)}{\partial\theta_m}\sum_{n',k'}\left[\frac{\partial\alpha_{n',k'}(i)}{\theta_l}A(n',k',n,k,i+1) + \alpha_{n',k'}(i)\frac{\partial A(n',k',n,k,i+1)}{\theta_l}\right]$$
$$+b_{n,k}(i+1)\sum_{n',k'}\left[\frac{\partial^2\alpha_{n',k'}(i)}{\partial\theta_l\partial\theta_m}A(n',k',n,k,i+1) + \frac{\partial\alpha_{n',k'}(i)}{\partial\theta_l}\frac{\partial A(n',k',n,k,i+1)}{\partial\theta_m}\right.$$
$$\left. + \frac{\partial\alpha_{n',k'}(i)}{\partial\theta_m}\frac{\partial A(n',k',n,k,i+1)}{\partial\theta_l} + \alpha_{n',k'}(i)\frac{\partial^2}{\partial\theta_k\partial\theta_m}A(n',k',n,k,i+1)\right]$$

In order to compute the derivative of these quantities with respect to N, which is a discrete parameter, binomial distributions were approximated by a normal distribution having the same mean and variance.
